# A review of size engineering-enabled electrocatalysts for Li–S chemistry

**DOI:** 10.1039/d1na00522g

**Published:** 2021-08-10

**Authors:** Xi Zhang, Yaping Zhang, Xijun Wei, Chaohui Wei, Yingze Song

**Affiliations:** State Key Laboratory of Environmental-Friendly Energy Materials, School of Materials Science and Engineering, Southwest University of Science and Technology Mianyang Sichuan 621010 P. R. China yzsong@swust.edu.cn; College of Energy, Jiangsu Provincial Key Laboratory for Advanced Carbon Materials and Wearable Energy Technologies, Soochow University Suzhou 215006 P. R. China chwei@suda.edu.cn

## Abstract

Li–S batteries (LSBs) have received extensive attention owing to their remarkable theoretical capacity (1672 mA h g^−1^) and high energy density (2600 W h kg^−1^), which are far beyond those of the state-of-the-art Li-ion batteries (LIBs). However, the retarded sulfur reaction kinetics and fatal shuttle effect have hindered the practical implementations of LSBs. In response, constructing electrocatalysts for Li–S systems has been considered an effective strategy to date. Particularly, size engineering-enabled electrocatalysts show high activity in the sulfur redox reaction, considerably contributing to the latest advances in Li–S system research. In this tutorial review, we provide a systematic summary of nano- to atomic-scale electrocatalysts employed in Li–S chemistry, aiming at figuring out the working mechanism of size engineering-enabled electrocatalysts in the sulfur redox reaction and guiding the rational construction of advanced LSBs toward practically viable applications.

## Abstract 2

We offer a systematic summary of electrocatalysts from nano to atom scale, aiming at revealing the working mechanism of size engineering-enabled electrocatalysts in sulfur redox reaction and guiding the rational construction of Li–S batteries.

## Graphical abstract

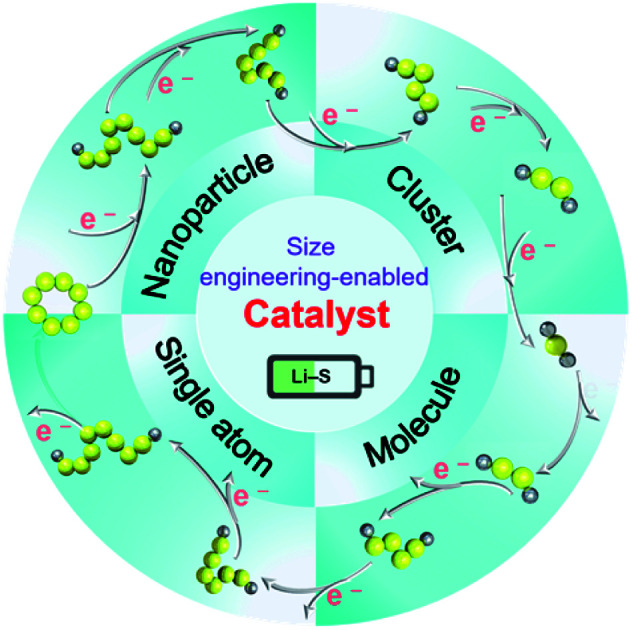

## 1. Introduction

The booming electrical vehicles and portable electronic equipment have urged the demand for advanced energy storage systems with high energy densities and low cost. Before the concept of rechargeable Li–S batteries (LSBs), Li-ion batteries (LIBs) were regarded as the key energy storage system to meet practical needs. Unfortunately, the energy density of LIBs is approaching the theoretical limit, probably because of failing to store large-scale energy for future applications.^1^ LSBs can serve as one of the most promising alternatives to LIBs due to their high theoretical specific capacity and outstanding energy density. Furthermore, the abundance and non-toxic nature of sulfur endow LSBs with low manufacturing cost and favorable environmental friendliness, respectively.^2,3^ However, several other obstacles hinder the commercialization of LSBs. The shuttle effect, which is caused by polysulfide (PS) migration between the cathode and the anode, leads to irreversible sulfur loss and anode corrosion. Other than that, the insulating nature of sulfur and discharge products (Li_2_S/Li_2_S_2_) give rise to the retarded electrochemical reaction redox kinetics, as well as the low utilization efficiency of active material. The combination of these issues results in the low discharge capacity and inferior cycling stability.^4,5^

To address the above-mentioned issues, various mediators involving carbon-based materials^2^ and metal compounds^6–8^ have been extensively used as promotors in Li–S systems. Ideal promotors are expected to synergize the remarkable immobilization ability for PS shuttle and high electrocatalytic activity for better sulfur redox reaction kinetics. The current evidences corroborate that it is essential to build a smooth PS adsorption–diffusion–conversion process on the surface of mediators for LSBs. The PS adsorption ability of these promotors mainly lies in their polar surfaces, which has been widely investigated. Particularly, rationalizing the electrocatalysis of Li–S chemistry is still waiting to be throughout long-term endeavors from the activity design of electrocatalysts. In this regard, effective strategies such as interface,^9^ defect,^10^ and template engineering^11^ have emerged. These strategies have managed to enhance the activity of the electrocatalysts in Li–S chemistry to a certain extent. However, it is urgent to further enrich the design routes targeted at highly active electrocatalysts. Since the size of the electrocatalysts plays a pivotal role in determining the activity, size engineering on electrocatalysts by reducing their sizes has been proposed to rationalize the electrocatalysis of LSBs.

Downsizing the particles from the nanoscale^12,13^ to the cluster,^14^ molecule,^15^ and even atomic levels^16^ leads to an augmented number of active sites or optimized coordination configurations, which is favorable to boosting the sulfur redox reaction efficiency and thereby contributes to the high capacity and long lifespan of LSBs. This review focuses on the recent advances and prospects on electrocatalyst designs from the nano to atom scale toward high activity in Li–S chemistry, aiming at offering rational strategies and new insights for improving the electrocatalysis of Li–S systems.

## 2. Electrochemistry and challenges of LSBs

**Fig. 1:**
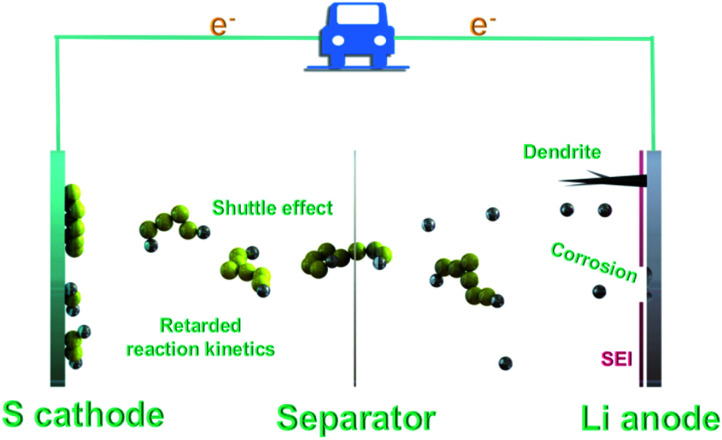
Schematic of the construction of routine LSBs.

In a working LSB, solid sulfur molecules (S_8_) firstly dissolve to form high-order Li_2_S_8_ molecules and then are reduced to Li_2_S_6_ and Li_2_S_4_, contributing to the first typical discharge plateau at approximately 2.4 V (Fig. 1). The soluble Li_2_S_4_ intermediates continue to generate the low-order insoluble compounds Li_2_S_2_ and Li_2_S, giving rise to the second plateau at around 2.1 V, which contributes to three-quarters of the total capacity.^17^ The multi-step phase conversions from S_8_ to Li_2_S accompanied by multi-electron chemistry cause the notorious shuttle effect and slow the sulfur redox reaction kinetics, thus resulting in irreversible sulfur loss and continuous anode corrosions, as well as limited discharge depth and rate. Notably, the issue of retarded sulfur redox reaction kinetics mainly results from the insulating nature of sulfur and Li_2_S, viscosity change in the electrolyte, slow solid-state diffusion and Li_2_S precipitation on the lithium anode. Along this line, the problem of kinetics is complex and can be affected almost by the whole electrochemical reaction procedure. In particular, these issues become more formidable under the practical scenario of high sulfur loading. Therefore, the investigation of reaction kinetics can break the gap between fundamental exploration and real implementation for LSBs. Additionally, lithium dendrites also pose a grand threat to the electrochemical performance and safety of LSBs. Scientific and technological viewpoints have been proposed by applying size engineering-derived promotors, which are of utmost importance to probing the feasibility of accelerating Li–S chemistry and unravelling the underlying electrocatalytic mechanism. In this light, the focus will continue to be centred on the development of new routes for the synthesis of active electrocatalysts.

## 3. Size engineering-enabled electrocatalysts for Li–S chemistry

The afore-mentioned issues limit the electrochemical performance of LSBs, leading to a sluggish commercialization process. The use of electrocatalysts in LSBs has been proposed as an effective strategy to address these challenges. Generally, the activity of electrocatalysts is largely restricted by their size. In this light, size engineering has been used as an emerging strategy to upgrade the activity of electrocatalysts by minimizing their sizes from the nanoscale to clusters, molecules and even the atom level. In this section, this review summarizes the progress of size engineering-enabled electrocatalysts, including nanoparticles, clusters, molecules and single metal atoms.

A decrease in the size of electrocatalysts usually leads to larger surface areas or uniformly dispersed single atoms, implying an increased number of active sites or high atom utilization efficiency, respectively. In electrocatalysts, both the abundance of active sites and high atom utilization efficiency manifest superior chemical activity, mainly pertaining to outstanding PS-anchoring ability and remarkable electrocatalytic capability for improved sulfur redox reaction kinetics. Therefore, the active site number is usually evaluated effectively to guarantee the chemical activity of the electrocatalysts. Further, the focus must be on the balance between the large surface area and single atom utilization efficiency, PS adsorption ability, Li^+^-ion migration and electron conductivity to attain high electrocatalytic activity.

### 3.1 Milestones of electrocatalysts from nano to atomic scales

**Fig. 2:**
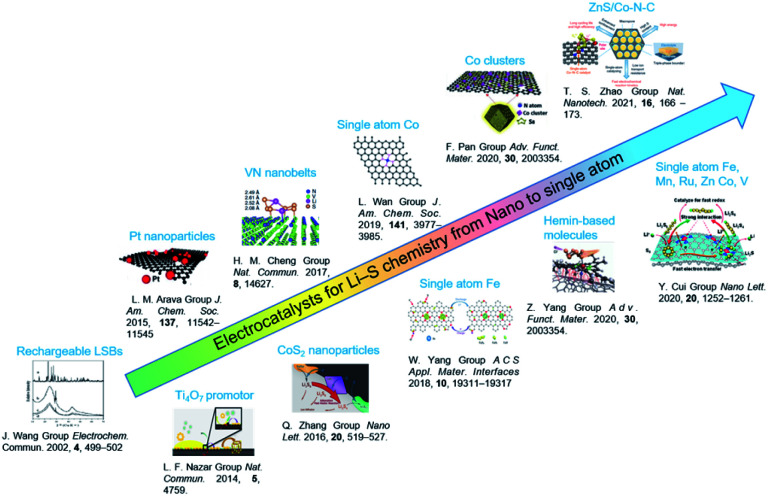
Electrocatalysts for LSBs from nano to single atom levels. Images reproduced with permission as follows: “Rechargeable LSBs”.^26^ Copyright 2002, Elsevier. “Ti_4_O_7_ promotor”.^19^ Copyright 2014, Nature Publishing Group. “Pt nanoparticles”.^27^ Copyright 2015, American Chemical Society. “VN nanobelts”.^28^ Copyright 2017, Nature Publishing Group. “CoS_2_ nanoparticles”.^29^ Copyright 2016, American Chemical Society. “Single atom Co”.^30^ Copyright 2019, American Chemical Society. “Single atom Fe”.^31^ Copyright 2019, American Chemical Society. “Co clusters”.^32^ Copyright 2020, Wiley-VCH. “Hemin-based molecule”.^15^ Copyright 2020, Wiley-VCH. “ZnS/Co–N–C”.^33^ Copyright 2020, Nature Publishing Group “Single atom Fe, Mn, Ru, Zn, Co, V”.^34^ Copyright 2020, American Chemical Society.

In recent years, electrocatalysts have been widely applied to improve the utilization and cyclic performance of LSBs.^14,15,18^ Since the concept of rechargeable LSBs was proposed in 2002,^26^ fruitful achievements have been made in size engineering-enabled electrocatalysts through constant explorations from fundamental to practical investigations (Fig. 2). A pioneering work by Nazar and co-workers in 2014 reported that Magneli-phase Ti_4_O_7_ offered an intrinsic polar surface to trigger interface-mediated redox reactions of PSs.^19^ Following this research, various nanocatalysts, including oxides,^20^ sulfides,^21^ nitrides,^22^ and phosphides,^23^ were used to improve the sulfur redox reaction kinetics. To further elevate their activity in Li–S chemistry, the sizes of the nanocatalysts were further decreased to cluster, molecule and single-atom levels. Zhang *et al.* proposed the application of atomic catalysts in heterogeneous catalysis in 2011.^24^ The model of single-atom catalysts (SACs) was conceived to make full use of the local metal atoms to realize fast electrochemical reactions in LSBs. For instance, SAFe was found to exhibit lower delithiation energy barriers, resulting in fast PS conversion and higher capacity.^25^ It is worth noting that SACs represent a new frontier in offering high-efficiency Li–S chemistry owing to their nearly 100% atom utilization efficiency and tunability.

### 3.2 Mechanism of electrocatalysts from nano to atomic levels

Electrocatalysts are extensively applied to deal with the stepwise reduction of S_8_ to soluble Li_2_S_4_ and further conversion to low-order insoluble Li_2_S. Particularly, the conversion of soluble Li_2_S_4_ to Li_2_S, which is called “Li_2_S nucleation and growth”, contributes to almost three-quarters of the total capacity, manifesting the critical electrochemical reaction steps.^35,36^ It is worth noting that this procedure refers to the formation of high-viscosity Li_2_S_4_ and the liquid–solid–solid conversion,^37,38^ implying that it is the speed-determining step of the electrocatalytic reaction. When the electrocatalyst is incorporated into the cathode of LSBs, the adsorption and electron transfer processes can be altered.^39^ The soluble PSs can be trapped by polar electrocatalysts followed by continuous and gradual conversion to solid Li_2_S during the reduction process.^40^ Therefore, the electrocatalytic mechanism can be explored by mainly investigating these two steps.

#### 3.2.1 Nanoparticle catalysts

**Fig. 3:**
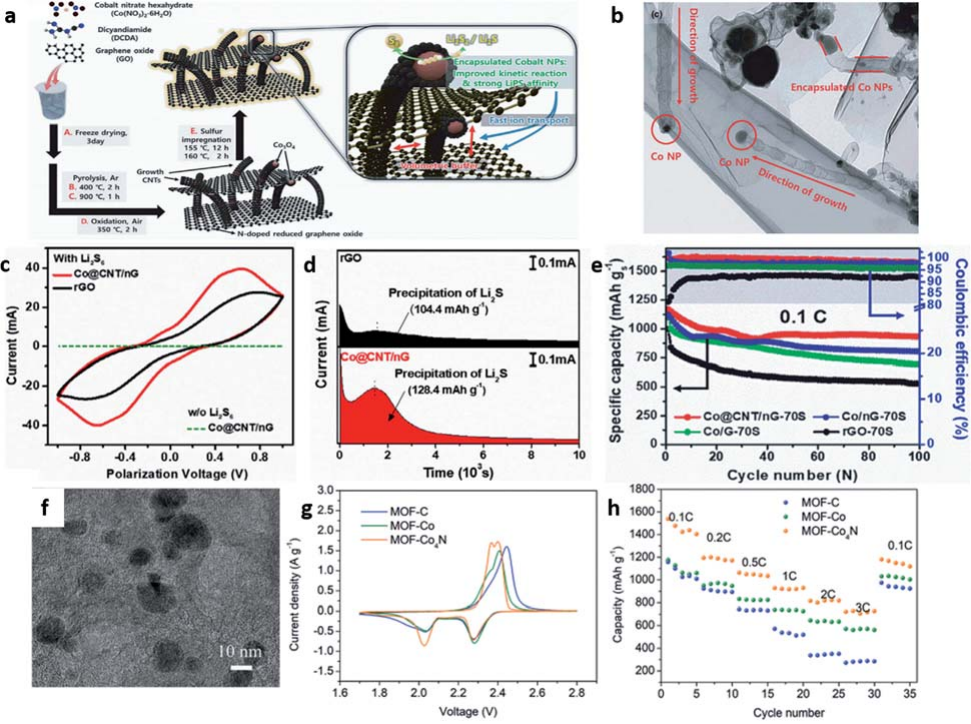
(a) The schematic illustration for the synthetic procedures and functional roles (zoomed image) of Co@CNT/nG-70S. (b) High-magnification TEM images of grown CNT of Co@CNT/nG. (c) CV curves of symmetric cells with Co@CNT/nG and rGO. (d) Potentiostatic discharge curves of Li_2_S_8_ solution at 2.06 V of Co@CNT/nG and rGO. (e) Cycling performance and coulombic efficiencies at 0.1C.^42^ Copyright 2021, Wiley-VCH. (f) TEM image of MOF-Co_4_N. (g) The second cycle of CV. (h) Rate performance of MOF-C, MOF-Co, and MOF-Co_4_N.^43^ Copyright 2019, Wiley-VCH.

Based on the in-depth research of LSBs, researchers have found that metal compounds possess strong chemical adsorption ability and different catalytic effects towards PSs, benefiting the effective anchoring of PSs and promoting their conversion.^41^ However, it is relatively difficult for electrocatalysts of overly large sizes to endow the cathode with high sulfur loadings and outstanding energy density. Hence, the size design for electrocatalysts must be optimized. Yeon *et al.* presented a hybrid for LSBs with multidimensional architecture by encapsulating cobalt oxide nanoparticles into carbon nanotubes that interspersed N-doped reduced graphene oxide networks (Fig. 3a and b).^42^ The electrocatalytic ability of the homogeneously distributed Co_3_O_4_ nanoparticles could be verified (Fig. 3c and d). As a result, a high initial capacity of 1193.1 mA h g^−1^ at 0.1C was finally achieved (Fig. 3e). Xiao *et al.* designed a two-dimensional (2D) N-doped carbon structure as the cathode, which had the Co_4_N nanoparticles stemming from the metal–organic framework (MOF) uniformly embedded on carbon cloth (Fig. 3f).^43^ The effective electrocatalytic activity in the PS redox reaction was demonstrated by the well-defined redox peaks in the CV curves (Fig. 3g) and the favorable rate performance (Fig. 3h).

Conductive and nonconductive promotors usually present distinct mechanisms. In detail, the effect of nonconductive mediators, such as oxides, on Li–S chemistry relies on the adsorption and diffusion of PSs on their surface due to the limited electron conductivity. Conductive mediators show superior electrocatalytic activity on account of their complete catalysis throughout the whole electrochemical reaction process. Although size engineering can be applied to both types of electrocatalysts to form more active sites, it is very challenging to quantify the elevation of activity according to the scale change.

#### 3.2.2 Cluster catalysts

**Fig. 4:**
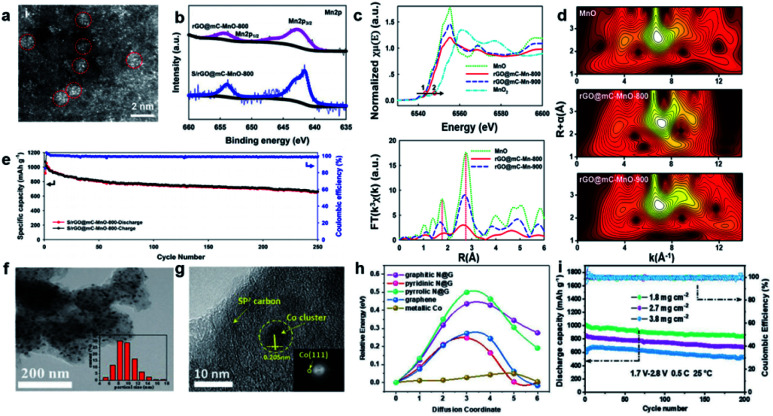
(a) HAADF-STEM images of MOC-embedded 2D rGO@mC nanosheets. (b) The Mn2p XPS spectra of rGO@mC-MnO-800 and S/rGO@mC-MnO-800. (c) XANES and *k*_3_-weighted FT-EXAFS in *R* space. (d) Wavelet transforms of rGO@mC-MnO-800 and the reference samples, including MnO, MnO_2_, and rGO@mC-MnO-900. (e) Rate capabilities of the cells assembled with S/rGO@mC-MnO-800 at various current densities.^46^ Copyright 2020, American Chemical Society. (f and g) SEM and HR-TEM of N-PC@uCo/S. (h) Calculated energy barriers for Li-ion diffusion. (i) Cycling capacity and coulombic efficiency of N-PC@uCo/S with the sulfur loadings of 1.8, 2.7, and 3.8 mg cm^−2^.^32^ Copyright 2020, Wiley-VCH.

Although nanoparticles have been demonstrated as an effective approach to realize advanced LSBs through enhancing chemical adsorption and promoting redox reaction kinetics,^43–45^ most of them are generally post-supported and thus exhibit low atom utilization efficiency. When the size of the nanoparticles is reduced to clusters, which contain ten to a few hundred metal atoms, the utilization of atoms significantly improves. Qiu *et al.* reported a single micelle-directed interfacial assembly strategy to realize the incorporation of sub-nanometric manganous oxide clusters (MOCs) into an N-doped mesoporous carbon single layer on graphene oxide.^46^ The magnified high-angle annular dark-field scanning transmission electron microscope (HAADF-STEM) image proved the successful incorporation of MOCs into the ordered N-doped mesoporous carbon (Fig. 4a). Moreover, the oxidation state of the Mn ions in MOCs could be identified by X-ray photoelectron spectroscopy (XPS) (Fig. 4b) and was in coordination with the X-ray absorption near-edge structure (XANES) (Fig. 4c and d). The as-prepared cathode displayed a reversible capacity of 684 mA h g^−1^ after 250 cycles at 2 A g^−1^ (Fig. 4e). The superior conductivity of the sub-nanometric MOCs effectively promoted the conversion reaction of sulfur to Li_2_S, and the mesoporous carbon substrate alleviated the volume change during the discharge–charge process. Furthermore, to verify the capability of the highly dispersed clusters in improving Li^+^-ion diffusion and PS absorption, Wang *et al.* used glucose-adsorbed MOF to obtain an N-doped porous carbon (N-PC) nanocage with uniformly dispersed cobalt catalysts (Fig. 4f and g).^32^ The synergistic structure was endowed with effective stress release, fast redox of PSs and strong physical/chemical adsorption, leading to a long cycle lifespan (86% capacity retention at 1C after 500 cycles) and favorable performance even at high a sulfur loading of 3.8 mg cm^−2^ (Fig. 4i). The density functional theoretical calculations further confirmed that the well-dispersed metal clusters significantly propelled the chemical absorption and conversion of PSs, thus boosting the specific capacity and rate performance (Fig. 4h).

#### 3.2.3 Molecule catalysts

**Fig. 5:**
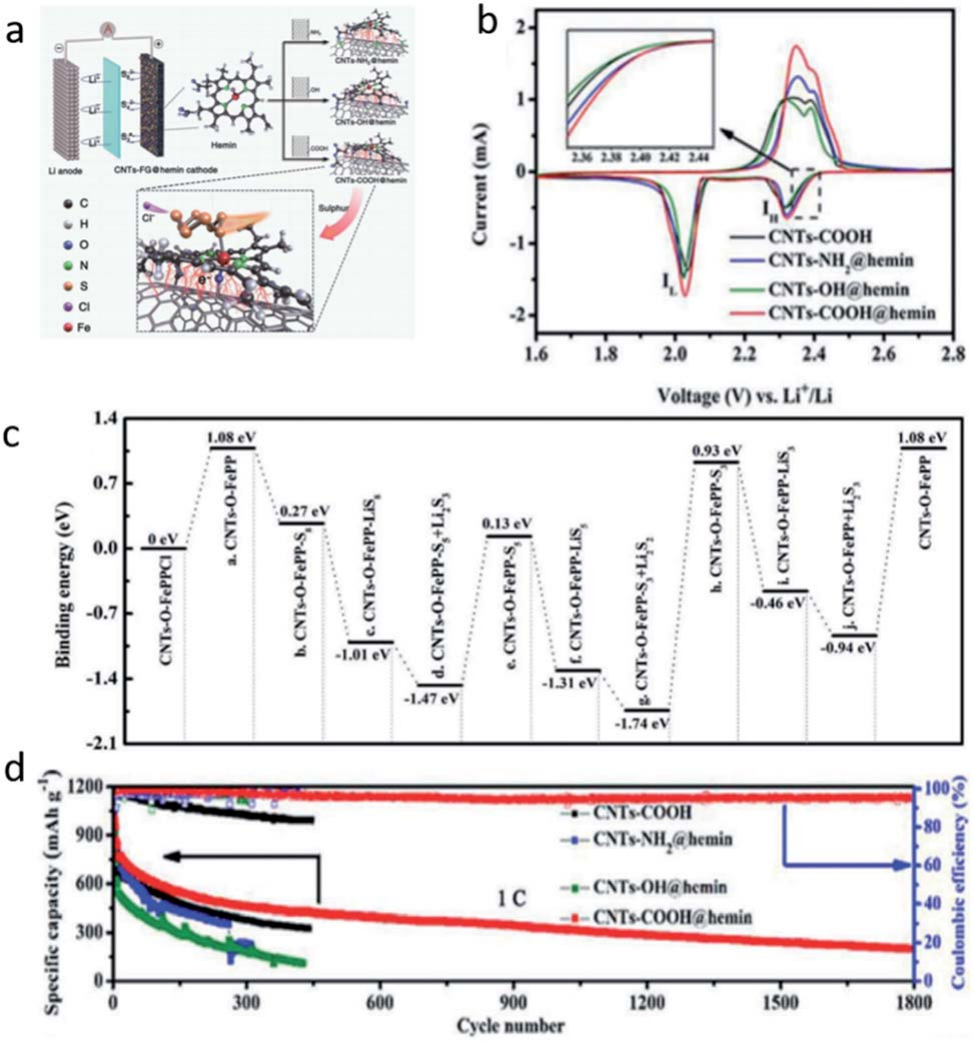
(a) Schematic configuration of an LSB based on three CNTs-FG@hemin cathodes (FG = NH_2_, OH, COOH), and the mechanism of PS adsorption at the CNTs–COOH@hemin cathode. (b) The second cycle of the CV profiles of the CNTs–COOH, CNTs–NH_2_@hemin, CNTs–OH@hemin, and CNTs–COOH@hemin cathodes. (c) Energy diagram for the conversion of PSs on CNTs–COOH@hemin. (d) Cycling stability and coulombic efficiency of the CNTs–COOH, CNTs–NH_2_@hemin, CNTs–OH@hemin, and CNTs–COOH@hemin cathodes at 1C.^15^ Copyright 2020, Wiley-VCH.

Despite some fruitful progress, the improved performance of these mediators with nano- or cluster sizes remains limited, especially at high areal sulfur loadings.^47^ Plenty of soluble PSs form and accumulate fast on the mediators, which along with the retarded reaction kinetics of Li–S chemistry lead to fast capacity decay.^48^ Along this line, the development of novel molecular mediators is desirable to accelerate PS conversion and mitigate the shuttle effect. Recently, Ding *et al.* presented a systematic design involving biomimetic molecule catalysts; hemin was grafted on functionalized carbon nanotubes to enhance the conversion of PSs for advanced LSB performance (Fig. 5a).^15^ The CNTs-COOH trapped the PSs in the organic electrolyte by forming π–π conjugation and coordinate bonds with hemin (Fig. 5c). Additionally, the CNTs-COOH@hemin showed high PS-capturing capability *via* the coordinated Fe(iii) complex containing the Fe–O bond, which enabled the faster conversion of long-chain S_8_^2−^ into S_3_^2−^ (or 
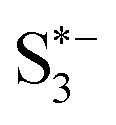
) during the discharge procedure in LSBs (Fig. 5b). Hence, an ultrahigh initial capacity of 1637.8 mA h g^−1^ at 0.2C and a low fading rate of 0.042% per cycle up to 1800 cycles at 1C were achieved (Fig. 5d). Lai *et al.* also developed a molecular catalyst, namely tris (4-fluorphenyl) phosphine (TFPP), as an interfacial mediator for longevous LSBs, which produced an impressive ultimate capacity of 545 mA h g^−1^ after 140 cycles at 5C with a high sulfur loading of 4.2 mg cm^−2^.^9^

#### 3.2.4 Single-atom catalysts

Since the catalytic performance is closely related to the number of active sites, further reduction in the size of electrocatalysts to the atomic scale is a promising approach to realize preferable activity. Due to the uniform distribution of single metal atoms, SACs can reach a 100% theoretical atomic utilization rate.^49,50^ SACs have been applied in various catalytic systems, which present superior catalytic performance, surpassing those of the traditional metal nanoparticles. However, due to the high surface free energy of the single metal centre, SACs are chemically unstable and have a tendency to aggregate into metal nanoparticles, thereby reducing the catalytic activity. Heteroatom-doped carbon substrates with large surface areas and high conductivity are usually selected as ideal substrates to stabilize SACs; especially, doped nitrogen atoms can readily coordinate with single metal atoms by forming M − N bonds.^51^

**Fig. 6:**
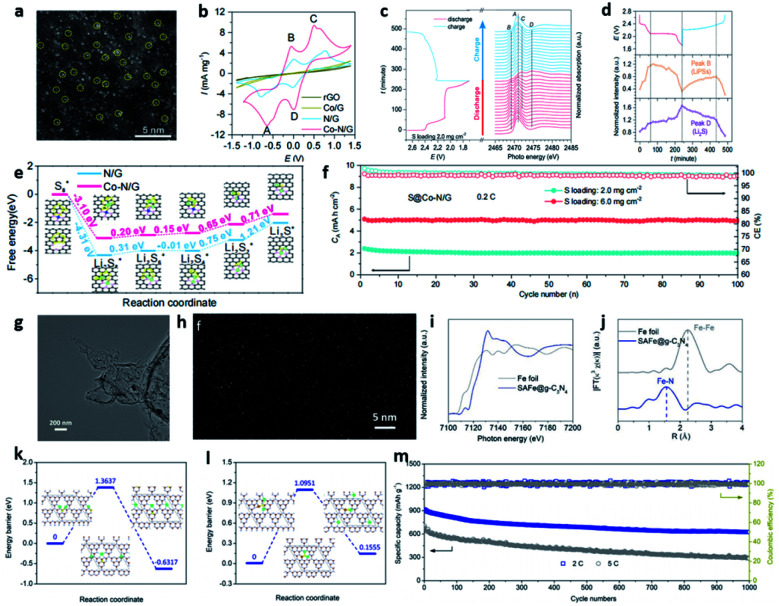
(a) HAADF-STEM images of Co–N/G. (b) Evolution of S K-edge XANES during electrochemical cycling. (c) Evolution of the intensities of peak B and peak D during electrochemical cycling. (d) CVs of the symmetric cells with Co–N/G, N/G, Co/G, and rGO as electrodes. (e) Energy profiles for the reduction of PSs on the N/G and Co–N/G substrates. The optimized adsorption conformations of the intermediate species on the N/G and Co–N/G substrates are shown in the insets. Energy profiles of the decomposition of Li_2_S clusters on N/G. (f) Cycling performance of the S@Co–N/G electrode with the areal S loadings of 2.0, 3.4 and 6.0 mg cm^−2^ at 0.2C.^30^ Copyright 2019, American Chemical Society. (g) TEM image of b-Fe@g-C_3_N_4_ materials. (h) HAADF-STEM image of the SAFe@g-C_3_N_4_ material showing single iron atoms. (i) Fe K-edge XANES spectra of Fe foil and SAFe@g-C_3_N_4_ material. (j) Fourier transform curves of the Fe K-edge EXAFS spectra of Fe foil and SAFe@g-C_3_N_4_. Energy profiles for (k) Li_2_S g^−1^-C_3_N_4_, and (l) Li_2_S/SAFe@g-C_3_N_4_. (m) The long-term stability of the device at the current rates of 2 and 5C.^25^ Copyright 2020, American Chemical Society.

Recently, Du *et al.* loaded SACo on N-doped graphene (Co–N/G) for a Li–S system (Fig. 6a).^30^ The Co–N–C coordination centres acted as dual-function electrocatalysts to accelerate the formation and decomposition of Li_2_S in the discharge and charge processes, respectively (Fig. 6b). The DFT calculations in Fig. 6e further indicate the lower Gibbs free energy of Li_2_S_2_ to Li_2_S conversion on Co–N/G than on N/G, indicating more favorable reaction thermodynamics. The *in situ* XANES in Fig. 6c and d further indicate that the introduction of SA on NG can facilitate the formation/decomposition of Li_2_S and Li_2_S_2_.^30^ Lu and co-workers devised a high-loading single metal atom material (SAFe@gC_3_N_4_) with an excellent catalytic activity that boosted the electrochemical conversion kinetics in LSBs (Fig. 6g–j).^25^ Owing to the strong coordination effect of the N sites, g-C_3_N_4_ was applied as a support for SAC with a high Fe atom content of 8.5 wt%. SAFe@gC_3_N_4_ could effectively suppress the shuttle effect of PSs and accelerate the conversion reaction, consequently reducing sulfur loss in the charge and discharge processes (Fig. 6k and l). These works offer new insights into the rational design of advanced SACs toward achieving high-performance LSBs. However, the single atom loading is low (<4.0 wt%), paving new development direction for SACs in Li–S chemistry.^52^

The interactions of well-dispersed single metal atoms with carbon substrates, mainly pertaining to the direct coordination of SA-C and indirect coordination, such as SA-N-C and SA-O-C, represent the active centers for electrocatalytic reactions. The difference between the metal atoms in SACs and the heteroatoms in mediators favor the high activity of SACs. The heteroatoms on the mediators can also serve as active sites for Li–S chemistry. Particularly, lithiophilicity, which is formed by the interaction between the heteroatoms and Li^+^-ions, can guide the sulfur redox reaction kinetics.^53^ However, the electrocatalytic activity of these heteroatoms cannot be compared with single metal atoms. This is because the metal atoms in SACs generally display unique electronic structures, unsaturated coordination environment and exclusive selectivity, leading to superior electrocatalytic activity in LSBs.

## 4. Conclusions and perspectives

The retarded reaction kinetics and notorious PS shuttling severely reduce the working efficiency and electrochemical performance of LSBs. Appropriate electrocatalysts that possess a strong affinity for PSs and impressive catalytic capability towards their conversion reactions are needed to boost the electrochemical performance of LSBs (Table 1). The disadvantages of size reduction include limited synthetic routes, agglomeration and dissolution of electrocatalysts in the electrolyte during long-term cycling. Therefore, stabilizing the small-sized electrocatalysts on selected substrates, such as carbon and metal compounds, has been corroborated as a preferred strategy. Moreover, the substrates can be further tuned into various structures with favorable surface areas to mitigate agglomeration or dissolving into the electrolyte, which ensures the high utilization efficiency of electrocatalysts. For instance, loading single atoms on carbon substrates with large surface areas does not only result in near 100% atom utilization efficiency but also optimizes the coordination configurations, thus making it an effective strategy to tackle these disadvantages.

**Table 1:** Comparison of the electrochemical performance of batteries based on electrocatalysts of various sizes

Types	Electrocatalysts	Areal loading of S (mg cm^−2^)	Capacities (mA h g^−1^) at various rate (C)	Cycle number	Capacity decay (per cycle)	Ref.
Nanoparticles	MOF-Co_4_N	1	745 mA h g^−1^ at 1C	400	0.043%	43
	Co@NHCRs	0.37	971 mA h g^−1^ at 0.5C	100	0.27%	54
	N–CN-750@Co_3_Se_4_-0.1	1.5 & 3.1	1150.3 mA h g^−1^ at 0.2C	800	0.067%	55
Clusters	N-PC@uCo	1.8–2.7 & 3.8	912 mA h g^−1^ at 1C	500	0.028%	32
	CE-NVO	6	686 mA h g^−1^ at 1C	200	0.1%	56
	rGO@mC-MnO-800	2.5 & 4.7	1535 mA h g^−1^ at 0.2C	100	0.36%	46
	S@Co/PNC	1.5	540 mA h g^−1^ at 1C	300	0.064%	57
	rGO−CNT/PW_12_	3.1 & 4.1	1145 mA h g^−1^ at 1C	150	0.084%	58
Molecules	CNTs-COOH@hemin	6.52	1637.8 mA h g^−1^ at 0.2C	1800	0.042%	15
	G@CB	4.2	1032 mA h g^−1^ at 0.2C	300	0.001%	59
	CNTs-S-TFPP	4.2	517 mA h g^−1^ at 5C	1000	0.042%	9
SACs	Co–N/C	2 & 6	926 mA h g^−1^ at 1C	500	0.053%	30
	Zn_1_–HNC	4.6 & 7.8	1225 mA h g^−1^ at 0.5C	100	0.009%	60
	SAFe@g-C_3_N_4_	3	1255 mA h g^−1^ at 0.2C	200	0.05%	25
	S@SA-Zn-MXene	1.7–5.3	802 mA h g^−1^ at 4C	400	0.03%	61
	Fe-PNC	1.3	1138.6 mA h g^−1^ at 0.1C	300	0.2%	31

The future applications of LSBs strongly depend on the progress of active electrocatalysts and the deep exploration of the underlying mechanisms. With the development of versatile size-engineering strategies, grand advances have been achieved in recent years.^25,43^ This tutorial review discusses the latest research achievements in terms of the electrocatalytic mechanisms in Li–S chemistry based on emerging electrocatalysts from the nano- to the atomic level and concludes with the key challenges and prospects in this field:

(i) Catalyst innovation: based on previous reports, an ideal electrocatalyst design involves an ample number of active sites, superb atom utilization efficiency, and high loading content, as well as remarkable conductivity. The activity of electrocatalysts in Li–S chemistry can be altered by delicate size engineering. However, newer electrocatalysts need to be further innovated by the continuous optimization of size engineering and the development of other new strategies.

(ii) Mechanism detection: an in-depth understanding of the mechanism of electrocatalysts is beneficial to further promote their activity. Emerging techniques, such as synchrotron radiation and neutron scattering, can record the real-time signals of PS intermediates in a working LSB, thus offering opportunities to understand the underlying electrocatalytic mechanism. Despite this, in view of their small sizes down to the atomic level, these electrocatalysts urgently call for advanced analytical techniques with higher resolution and wider detection range to achieve all-level surface and interface information for a clear interpretation of the electrocatalytic mechanisms.

## Conflicts of interest

There are no conflicts to declare.

## Supplementary Material
